# A Practical Approach to Malaria Diagnosis in Non-Endemic Regions: Evaluating Simple Clinical and Laboratory Predictors in Travelers Returning from Endemic Regions

**DOI:** 10.1186/s41182-025-00682-z

**Published:** 2025-06-17

**Authors:** Amal A. El-Moamly

**Affiliations:** Department of Medical Parasitology, Faculty of Medicine, Suez Canal University, Ismailia, Egypt

**Keywords:** Malaria, Diagnosis, Nonendemic, Imported, Laboratory tests, Clinical features, Diagnostic set, Predictors, Hypocholesterolemia, Leukopenia, Thrombocytopenia

## Abstract

**Background:**

Malaria, a life-threatening parasitic disease, remains a significant global health challenge. Malaria diagnosis in nonendemic regions can be challenging because of limited expertise and resources; rapid and accurate diagnosis is crucial for timely treatment and prevention of disease transmission. To improve diagnostic performance, this study aimed to evaluate the utility of clinical and laboratory parameters as predictors of malaria infection in patients presenting with fever after returning from malaria-endemic areas.

**Methods:**

A prospective observational hospital-based study with convenience sampling was conducted among febrile patients presenting to the emergency department in Riyadh city/Saudi Arabia with a history of travel to malaria-endemic regions. The detailed clinical information and laboratory parameters, including complete blood count, liver function tests, cholesterol, and lactate dehydrogenase, were collected. Malaria was confirmed by rapid diagnostic tests (RDTs) and microscopic examination of blood smears. The diagnostic accuracy of various clinical and laboratory predictors was assessed via sensitivity, specificity, positive predictive value, negative predictive value, and likelihood ratios.

**Findings:**

While no single clinical or laboratory predictor was sufficient to definitively diagnose malaria, a combination of these factors proved to be a valuable tool. Low cholesterol (<3 mmol/L) demonstrated high sensitivity, whereas low platelet count (<150 × 10^9^/L) exhibited high specificity. Elevated lactate dehydrogenase (>190 U/L) had the highest sensitivity but lower specificity. A combination of these laboratory markers, along with fever, vomiting, and chills, showed better performance in the diagnosis of malaria.

**Conclusion:**

This study highlights the potential of a simple, clinical approach to aid in the diagnosis of malaria in nonendemic settings. A combination of clinical features and laboratory tests can significantly improve diagnostic accuracy, particularly in resource-limited settings. Further validation studies are needed to refine and optimize this approach.

## Introduction

Malaria is one of the oldest of deadly enemies of humankind, and is still a major health problem for many countries. According to the World Health Organization’s (WHO) 2023 World Malaria Report [1], the number of malaria cases worldwide in 2022 reached 249 million—a concerning rise of five million compared with prepandemic projections. While malaria deaths steadily declined between 2000 and 2019, they reached 608,000 deaths in 2022—approximately 32,000 more than prepandemic levels, with children under five making up the majority. Approximately half of the world’s population is still at risk for contracting malaria [1].

In nonendemic regions, the number of imported malaria cases has increased, with mortality rates ranging from 3.8 to 20% [2]. The presence of endemic foci in different parts of the world, population movements, international travel, large governmental agricultural projects, and increased global warming all increase the number of imported cases, posing diagnostic challenges for healthcare providers with limited malaria expertise. As a result, malaria has been reported to be missed initially in up to 60% of patients seeking hospital consultation in nonendemic areas [3].

Early and accurate diagnosis is crucial for timely and effective treatment and prevention of further transmission. While microscopic examination of stained blood smears remains the gold standard, its sensitivity and specificity can be limited by factors such as parasite density and examiner experience [4]. In addition, the time-consuming nature of this method can hinder prompt diagnosis. While fluorescence microscopy can improve the sensitivity of microscopic examination, it does not increase specificity [5]. Although highly sensitive, molecular diagnosis by PCR is often impractical for routine diagnosis because of cost and technical requirements [6]. Rapid diagnostic tests (RDTs) offer a rapid, nonmicroscopic alternative, but their sensitivity can be limited, particularly in nonimmune populations with low parasite densities [7, 8].

To address these limitations, there is a need for simple, practical, and accessible diagnostic tools to aid in the identification of malaria cases, particularly in nonendemic settings. This study aimed to evaluate the utility of specific clinical and laboratory predictors in identifying malaria cases among febrile travelers returning from malaria-endemic regions to nonendemic settings. By identifying a combination of clinical and laboratory markers that can reliably predict malaria infection, we hope to improve diagnostic accuracy and facilitate timely and appropriate treatment.

Riyadh, the capital city of Saudi Arabia, where the study was conducted, is a nonendemic setting for malaria. It does not exhibit active mosquito-borne transmission due to unfavorable climatic conditions [9]. Malaria transmission in Saudi Arabia is confined primarily to endemic areas in southern and southwestern Saudi, particularly Jazan Province. However, the capital’s large expatriate population and frequent travel to and from endemic areas contribute to the occurrence of imported malaria cases. *Plasmodium falciparum* is the predominant species detected in Saudi Arabia. The pattern of malaria incidence in Riyadh is influenced by factors such as seasonal variations in endemic areas, travel patterns of individuals, and the prevalence of different *Plasmodium* species in those areas [10]. The annual incidence of positive blood smears among referred cases has varied, with peak incidence observed during specific seasons. The peak incidence typically occurred from February to April, which coincides with the rainy season in endemic areas. Most positive cases were among Saudi nationals and individuals from malaria-endemic countries [11]. According to the WHO World Malaria Reports between 2000 and 2010, the annual incidence of malaria cases in Riyadh ranged from 100 to 300 cases.

## Methods

### Study setting

The study was carried out at Al-Iman General Hospital in Riyadh city, Saudi Arabia, which is not endemic for malaria. The hospital has 215 beds and serves approximately 1 million people who live in the southern parts of the city, where socioeconomic conditions are generally unfavorable. The hospital serves as a secondary healthcare institution and a referral center, receiving patients referred from other city health centers in addition to offering primary care services in the emergency and outpatient departments. More than 130,000 patients of various nationalities, including those from malaria-endemic countries, visit the emergency department (ER) each year. The Central Laboratory in Riyadh serves as a reference laboratory for malaria diagnosis in the region and receives referrals from hospitals, health centers, and private clinics. There are other clinical laboratories that also examine blood samples for malaria in Riyadh; however, the Central Laboratory receives the majority of the cases in the area of coverage. Hospital referrals have recently decreased, most likely as a result of hospitals, including Al-Iman General Hospital, becoming more independent in their use of malaria microscopy and hiring more expert laboratory personnel. However, there are no appreciable shifts in the quantity of cases referred from private clinics and health centers [11].

### Study participants

#### Recruitment process

All eligible patients who presented to the emergency department of Al-Iman General Hospital, Riyadh, Saudi Arabia, between November 2002 and April 2004, were consecutively enrolled via a convenience sampling method. The inclusion criteria included all age groups, both males and females, a recent travel history to a malaria-endemic region, and the presence of fever and at least one of the following symptoms: chills, sweating, vomiting, headache, backache, jaundice, signs of central nervous system involvement, or anemia. Patients with a history of antimalarial therapy within the previous 2 weeks were excluded. Permission and ethical approval to conduct the study were obtained from the Ministry of Health authorities and the administrative board of Al-Iman General Hospital. Verbal informed consent was obtained from all eligible participants or their legal guardians, ensuring adherence to ethical guidelines. The participants are given an explanation of the study’s purpose, all procedures, the risks and benefits of each technique, and the advantages and disadvantages of participating in the study. To ensure the confidentiality and privacy of the participants, all individuals provided informed consent before any data were collected. All acquired data were anonymized to eliminate any personally identifiable information. Only authorized personnel can access safely stored data. Data analysis was conducted on aggregated data to protect participant confidentiality. No published reports or presentations contained personally identifiable information.

### Patient evaluation

**Fig. 1:**
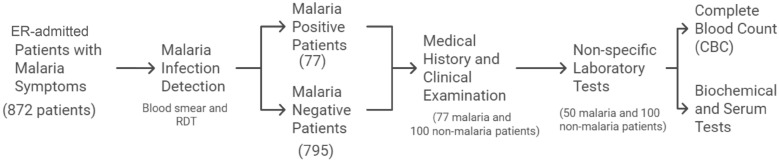
Flow chart of the study participants’ selection and procedures

A flow chart of the study participants that details the selection and procedures for the patients who are part of the study is shown in Fig. 1.

#### Medical history and clinical examination

Clinical and demographic data were collected through detailed medical histories and physical examinations and were obtained via personal interviews with the study participants via standardized data collection forms. This included travel history, use of antimalarial chemoprophylaxis or medications, a detailed history of the present illness, and a past history of malaria.

#### Laboratory assessment

Malaria was diagnosed by examining thick and thin blood smears, and with the OptiMal rapid diagnostic antigen-detection assay, an immunochromatographic test (ICT). Any discrepancies between the results of microscopic examination and ICT were interpreted as a warning to re-assess the stained blood smear [12]. The final results were reported according to the microscopic examination findings.

*Thick and thin peripheral blood smears to detect malaria parasites* One milliliter of venous blood was drawn. Capillary blood was taken by finger prick from patients who had negative venous samples and a high clinical suspicion of malaria. Thin and thick blood films were made shortly after collection as recommended by Wrhurst and Williams [13]. Thin blood films were used to identify malaria parasites, determine their species, and measure the level of parasitemia. Thick blood films, which are more sensitive, were used to confirm thin films and detect low levels of parasitemia, increasing the diagnostic accuracy. The thin film was spread with a tail that was at least 2 cm long, air-dried, and immediately stained with Wright’s stain without prior fixation. Buffered distilled water (pH 6.6) was used to highlight parasite inclusions in the RBCs, and the parasite species were identified. Thick films were stained with 20% Giemsa stain for 20 min with buffered distilled water (pH 7.2). Both thin and thick blood films were examined under an oil emersion 100× objective to detect sexual or asexual parasitic forms. If no parasites were visible in the thin film (shorter staining time than thick films), thick preparations (higher sensitivity than thin films) were then examined after they were dried and appropriately fixed. Blood smears were considered negative when no malaria parasites were found in 200 consecutive fields in the thick and thin films. If the first set of smears was negative and the patient had a high clinical suspicion of malaria, venous and/or capillary blood samples were taken at intervals of 6–18 h for at least 3 successive days before a diagnosis of malaria was ruled out [14].

*Estimation of the parasitemia level* To assess the disease burden and therapeutic success, the parasitemia level was determined according to the WHO [15]. A well-stained thin blood film was examined to determine the number of parasitized cells (not the individual parasites) seen in 10,000 RBCs (equal to approximately 40 monolayer cell fields) with a standard microscope and 100× oil-immersion objective. The average number of RBCs per microscopic field was used to estimate the parasitemia level in each patient. The approximate number of parasites in 1 μL of blood was calculated assuming that this volume of blood contains approximately 5 × 10^6^ RBCs. For example, a parasitemia level of 1% would represent an estimated 50,000 parasites/μL. This was corrected based on the total RBC number per microliter for each patient. The results were reported as the number of parasites per 100 RBCs (%) and per 1 μL of blood.

*Immunochromatographic assay* The OptiMal rapid diagnostic assay (DiaMed, under license from Flow Inc., Portland, OR, USA), which detects *Plasmodium*-specific lactate dehydrogenase (PLDH) enzymes, was used in conjunction with microscopic examination of blood films to increase the diagnostic accuracy for malaria. In accordance with the manufacturer’s instructions, 1 drop of whole blood was mixed with 2 drops of reagent A, which disrupts the erythrocytes and releases PLDH. The sample was allowed to migrate to the top of the strip. After 8 min, the strips were cleared with 2 drops of reagent B. A dark band on the strip indicated a positive reaction for any of the four major human malaria-causing species. The monoclonal antibodies at this site are panspecific for an enzyme common to all four species. If *Plasmodium falciparum* was present, a second band appeared at the site that contained the *P. falciparum*-specific monoclonal antibody. The control band at the top of the strip indicated that the test worked correctly.

##### Nonspecific laboratory tests

*Complete blood count (CBC)* Total and differential WBC and RBC counts, hemoglobin, hematocrit (HCT), mean corpuscular volume (MCV), mean corpuscular hemoglobin (MCH), mean corpuscular hemoglobin concentration (MCHC), and platelet count were recorded with an automated Peckman^®^ Coulter Counter. Differential WBC counts were confirmed with a manual counter.

*Biochemical serum parameters* Blood urea nitrogen (BUN), creatinine, total and direct bilirubin, lactate dehydrogenase (LDH), total cholesterol, sodium and potassium ions, random blood sugar, alkaline phosphatase (ALP), and alanine transaminase (ALT) and aspartate transaminase (AST) liver enzymes were assayed with an automated analyzer (Dimension AR DadeBehring^®^, Deerfield, IL, USA).

Severe malaria was defined as hyperparasitemia > 5%, severe anemia with hematocrit < 15 or hemoglobin < 5 g/dL, circulatory disturbance with shock or hypotension (<70 mmHg systolic blood pressure for adults), spontaneous bleeding (gums, nose, gastrointestinal tract), macroscopic hemoglobinuria, or impaired consciousness [16].

Different tests were performed in a blinded manner and were interpreted by different examiners independently from the results of other tests to ensure interrater reliability and assess the consistency of the data collected by different examiners. Other quality assurance and quality control measures in the laboratory and clinical departments are taken according to the Ministry of Health (MOH) guidelines. The data were collected through a combination of clinical assessment and laboratory investigations performed by experienced physicians and qualified laboratory personnel. The validity of the data collection tools was justified by either reciting credible sources such as the WHO or prior reliable research or by adhering to local health authorities’ recognized healthcare practice standards that are followed at the study site.

The diagnostic accuracy of the clinical and laboratory parameters that showed associations with malaria was assessed in patients with malaria and patients with other diagnoses who had complete clinical and laboratory evaluation data. Thin and thick blood film examination was considered the reference test, and the clinical and laboratory indicators were considered the index tests. True positive and negative cases were defined according to the results of the thick and thin blood smears. Patients with symptoms suggestive of malaria were tested in a blinded manner for the reference and index tests.

### Statistical analysis

The data were analyzed with SPSS version 19 software (Chicago, IL, USA). Descriptive statistics were compiled for demographic and clinical data. Odds ratios were used to estimate the strength of associations between malaria and different dependent variables such as hypocholesterolemia, thrombocytopenia, and land eukopenia. Chi-squared tests were used to test the independence of categorical variables to determine whether there was a statistically significant association between the presence of malaria and the occurrence of various clinical features, such as fever, vomiting, and chills. Independent *t* tests were used to compare differences in the mean of variables, such as platelet count and cholesterol, LDH, and bilirubin levels, between the malaria group and the other diagnoses groups. Fisher’s exact test and the Mann‒Whitney *U* test were used when appropriate. Parametric and nonparametric tests were chosen according to the normality of the population data. To assess the normality of the data, the Shapiro‒Wilk test was used. For clinical and laboratory parameters associated with malaria, sensitivity, specificity, positive predictive value (PPV), negative predictive value (NPV), positive likelihood ratio (+LR), negative likelihood ratio (−LR), and accuracy were calculated. The PPV, NPV, and accuracy were estimated based on the assumed prevalence of the disease. Diagnostic accuracy tests were performed via a 2 × 2 table with 77 true positive and 100 true negative results for malaria (for clinical parameters) and with 50 true positive and 100 true negative results (for laboratory parameters) as determined by the gold standard. 95% confidence intervals (95% CI) were estimated, and *p* values <0.01 were considered statistically significant. A nonparametric correlation test (Spearman’s correlation) was used to assess the correlation between the parasitic index and the mean platelet count, as well as between the platelet count and disease duration, in malaria patients. Missing data were handled via the complete case analysis technique, excluding cases with any missing data. Sensitivity analyses were performed to evaluate the impact of missing data on the study’s findings. To minimize data entry errors, double data entry was performed and discrepancies were resolved through cross-checking with the original medical forms.

The following formulas were used to calculate the diagnostic performance of various markers:

$${\text{Sensitivity}} = ({\text{True}}\,{\text{Positives}})/({\text{True}}\,{\text{Positives}} + {\text{False}}\,{\text{Negatives}})$$

$${\text{Specificity}} = ({\text{True}}\,{\text{Negatives}})/({\text{True}}\,{\text{Negatives}} + {\text{False}}\,{\text{Positives}})$$

$${\text{Positive}}\,{\text{Predictive}}\,{\text{Value}} = ({\text{True}}\,{\text{Positives}})/({\text{True}}\,{\text{Positives}} + {\text{False}}\,{\text{Positives}})$$

$${\text{Negative}}\,{\text{Predictive}}\,{\text{Value}} = {\text{(True}}\,{\text{Negatives)}}/{\text{(True}}\,{\text{Negatives}} + {\text{False}}\,{\text{Negatives)}}$$

$${\text{Positive}}\,{\text{Likelihood}}\,{\text{Ratio}} = {\text{Sensitivity}}/{(}1 - {\text{Specificity)}}$$

$${\text{Negative}}\,{\text{Likelihood}}\,{\text{Ratio}} = {(}1 - {\text{Sensitivity)}}/{\text{Specificity}}$$

$${\text{Accuracy}} = ({\text{True}}\,{\text{Positives}} + {\text{True}}\,{\text{Negatives}})/{\text{(True}}\,{\text{Positives}} + {\text{True}}\,{\text{Negatives}} + {\text{False}}\,{\text{Positives}} + {\text{False}}\,{\text{negatives)}}$$

## Results

### Baseline characteristics of the study participants

The study population included 872 patients who returned from different malaria-endemic regions with symptoms suggestive of malaria. Among them, 77 (9%, 95% CI 0.07, 0.11) had malarial infection as detected by microscopic examination of thin and thick blood smears and by ICT. The patients infected with malaria were from Saudi Arabia (64.9%), Sudan (24.7%), Pakistan (6.5%), India (2.6%), and Yemen (1.3%). The predominant malaria species varied significantly by nationality, with *P. falciparum* most common in Saudis and *P. falciparum* and *P. vivax* occurring with equal frequency in Sudanese individuals. *Plasmodium vivax* was the primary species in Pakistani, Indian, and Yemeni individuals (Table 1). All malaria-infected Saudi patients in this study had a history of travel to the southwestern region of the country (Jazan area). The peak incidence in Saudi patients was from December to March, which we attributed to the pattern of the rainy season in southern areas, which is followed by peak transmission of the disease [10]. Many Saudi patients were infected upon visiting Jazan for the first time for brief periods (2 or 3 days), reflecting the high transmission rate in this area. Patients who traveled outside the country were recorded throughout the year. In this study, foreign patients were infected with *Plasmodium* species, which represent the predominant species in their endemic home countries.

**Table 1:** Malaria species in the study population

Nationality	P.f No. (%)	P.v No. (%)	P.f & P.v No. (%)	Total No
Saudi	47 (94)	3 (6)	0	50
Sudanese	8 (42)	8 (42)	3 (16)	19
Pakistani	0	5 (100)	0	5
Indian	0	2 (100)	0	2
Yemeni	0	1 (100)	0	1
Total	55	19	3	77

*P.f Plasmodium falciparum*, *P.v Plasmodium vivax*

According to the parasitological assessment, patients were divided into two groups to compare their clinical and laboratory data: those with malaria and those with a different diagnosis (Tables 2, 3). A detailed summary of the baseline demographic and clinical characteristics of the two groups is shown in Table 2. Data in this study were available for 77 patients with malaria and 100 patients with other diagnoses (with the exception of laboratory parameters, which were available for only 50 malaria patients). The following diagnoses were assigned to the malaria-free group: septicemia (18%), hemolytic crisis due to sickle cell disease, thalassemia or G6PD deficiency (18%), infective febrile diarrhea (9%), lower respiratory tract infection (10%), upper respiratory tract infection (6%), urinary tract infection (4%), typhoid fever (3%), and meningitis (3%). Tuberculous meningitis, hemorrhagic fever, and autoimmune hemolytic anemia were diagnosed in 1 patient each. For the remaining patients, the final diagnosis was unknown due to failure of follow-up or for other reasons. Sixty-three of the 77 malaria patients had mild malaria, while 14 had severe malaria. The mean parasite density in the study participants was 1.9 ± 4.4% for *P. falciparum* (range 0.01–30%), 0.7 ± 0.6% for *P. vivax* (range 0.05–0.06%), and 3.3 ± 2.0% for mixed *P. falciparum* and *P. vivax* (range 1.0–4.4%) (*p* < 0.01).The mean parasite density was lower in semiimmune patients (17% of the malaria-infected group who had a past history of malaria infection) than in nonimmune patients who denied any history of malaria infection (0.6 ± 0.1% vs. 2.0 ± 0.9%) (*p* < 0.01, 95% CI −2.82, −0.83). The presentation of the disease was less severe in semiimmune patients than in nonimmune patients (22% vs. 0%, respectively), who had severe malaria. Patients in this study sought medical care after a duration of illness ranging from 3 to 7 days. All malaria patients recovered after treatment and were discharged when thin and thick blood films were free of asexual parasitic forms [17] after an admission period of no longer than 1 week.

**Table 2:** Baseline demographic and clinical characteristics of patients with malaria and other diagnoses

Characteristic	Malaria (77 patients)	Other diagnoses (100 patients)
Male/Female no. (ratio)	59/18 (3.3:1)	73/27 (2.7:1), *p* > 0.01
Age: mean (range) (years)	21.8 (2–45)	31.2 (3–95), *p* < 0.01
Days (mean) from return to Riyad to first appearance of symptoms (range)	*P.f P.v* (8.9) (59.1) (1–30) (7–300) *p* < 0.001	(1245) (> 1 year)
Past history of malaria: no./respondents (%)	13/77 (16.9%)	1/100 (1%), *p* < 0.01
*Signs & symptoms: no./respondents (%)*		
Fever	73/77 (94.8)	95/100 (95), *p* < 0.01
Chills	61/77 (79.2)	15/100 (15), *p* < 0.01
Diarrhea	12/77 (15.6)	11/100 (11), *p* > 0.01
Vomiting	55/77 (71.4)	22/100 (22), *p* < 0.01
Jaundice or dark urine	33/77 (42.9)	1/100 (1), *p* < 0.01
Bleeding	8/55 (14.6)	NDA
CNS symptoms (coma, convulsions, meningeal irritation)	6/77 (7.8)	5/100 (5), *p* > 0.01
Headache	63/77 (81.8)	35/100 (35), *p* < 0.01
Cough	4/77 (5.2)	15/100 (15), *p* > 0.01
Enlarged spleen	35/77 (45.5)	NDA
Enlarged liver	27/77 (35.1)	NDA
Mean temperature °C (± SD)	39.2 (± 0.3)	38.4 (± 0.5), *p* < 0.01
*No. patients with temperature (°C)*		
<37.5	1/77	2/100 (LR = 0.6)
37.5 to <38	2/77	35/100 (LR = 0.1)
38 to <38.5	3/77	42/100 (LR = 0.1)
38.5 to <39	25/77	12/100 (LR = 2.7)
≥39	46/77	9/100 (LR = 6.6)
Fever ≥ 38.5 °C: no./respondents (%)	71/77 (92)	21/100 (21%), *p* < 0.01

*NDA* no full data available, *LR* likelihood ratio

**Table 3:** Results of nonspecific laboratory tests in patients with malaria and other diagnoses

Lab test (normal range)	Malaria (50 patients) Mean ± SD (range)	Other diagnoses (100 patients) Mean ± SD (range)	*p* value
*WBCs*			
Total: 3.5–10 × 10^9^/L	5.91 ± 2.98 (2.5–16)	11.26 ± 4.5 (2.9–22.1)	<0.01
Neutrophils: 40–75%	48 (12–75)	71 (39–94)	<0.01
Lymphocytes: 20–45%	50 (25–87)	25 (6–53)	<0.01
Monocytes: 2–10%	1.1 (0–2.2)	1.3 (0–2)	>0.01
Eosinophils: 1–6%	1.5 (0–2)	0.9 (0–6)	>0.01
Hb concentration (M: 13–18; F: 12–16 g%)	10.7 (5.5–16.1)	11.9 (3.2–15.3)	>0.01
RBC count (×10^12^) (M: 4.5–6.5; F: 4–5/L)	4.02 ± 2.01 (1.9–6.3)	3.9 ± 0.5 (1.01–5.5)	>0.01
Hct (M: 40–54; F: 37–47)	31.3 ± 5.2 (15.6–47.1)	35.1 ± 4.7 (10.2–47)	>0.01
MCV (86–96 fL)	77.9 ± 9.1 (59–90.6)	84.3 ± 8.7 (65–101)	>0.01
MCH (27–32 pg)	24.8 ± 3.0 (18.2–29.5)	28.4 ± 3.1 (21–35.3)	>0.01
MCHC (30–35 g/dL)	32.2 ± 1.4 (24.3–35)	33.8 ± 1.5 (32–37)	>0.01
Platelet count (×10^9^) (150–400/L)	114.75 ± 67.46 (21–288)	232.66 ± 93.9 (87–279)	<0.01
Total cholesterol (2.5–6.4 mmol/L)	2.19 ± 0.56 (1.5–5)	4.15 ± 1.17 (1.2–5.8)	<0.01
LDH (100–190 U/L)	361.24 ± 122.16 (193–644)	200.64 ± 73.86 (44.3–622)	<0.01
Total bilirubin (<17 μmol/L)	66.73 ± 29.63 (15–335.8)	33.91 ± 28.27 (8–113)	<0.01
Direct bilirubin (<4.5 μmol/L)	67 ± 13 (2.6–265.9)	11.8 ± 10.1 (1.2–109)	>0.01
Creatinine (53–123 μmol/L)	82.9 ± 17.1 (4–249)	116.8 ± 24.4 (33–106)	>0.01
BUN (1.7–83 mmol/L)	15.9 ± 4.5 (2.2–27.8)	23.6 ± 6 (2.8–106)	>0.01
Sodium (133–152 mmol/L)	127.8 ± 7.1 (124–142)	138.7 ± 5.2 (134–141)	>0.01
Potassium (3.5–5.6 mmol/L)	3.8 ± 2 (3.2–4.6)	4.2 ± 1.1 (3.9–5)	>0.01
ALT (<40 U/L)	46.2 ± 2.2 (32–120)	41.5 ± 1 (30–150)	>0.01
AST (<40 U/L)	35.6 ± 3.4 (28–99)	41 ± 1.1 (39–43)	>0.01
Alkaline phosphatase (39–117 U/L)	138.7 ± 17 (44–198)	152.8 ± 23.2 (33–200)	>0.01
Amylase (<220 U/L)	37 ± 9.4 (32–55)	42 ± 9.3 (35–60)	>0.01

### Association of clinical features with malaria diagnosis

*Mean body temperature* On admission, the mean body temperature was higher in malaria-infected patients than in those with other diagnoses (*p* < 0.01) (Table 2). Ninety-two percent (71/77) of patients with malaria had a fever of ≥38.5 °C versus 21% (21/100) in the malaria-free group (*p* < 0.01) (Table 2). The cutoff temperature (≥38.5 °C) was selected based on the calculated likelihood ratios (Table 2) for further assessment of the association between temperature and a diagnosis of malaria. There was a positive association between fever ≥ 38.5 °C and a diagnosis of malaria (OR = 45, *p* < 0.01) (Table 4).

**Table 4:** Diagnostic performance of malaria predictors

Predictor	OR	Sensitivity% (95% CI)	Specificity% (95% CI)	PPV% (95% CI)	NPV% (95% CI)	+ LR	−LR	Total accuracy (95% CI)
WBC < 8 × 10^9^/L	3.6	69 (0.58–0.79)	62 (0.52–0.72)	15 (0.12–0.19)	95 (0.93–0.97)	1.8	0.5	63 (55–70)
Low platelets	20	71 (0.61–0.82)	89 (0.83–0.95)	39 (0.27–0.53)	97 (0.96–0.98)	6. 5	0.3	87 (82–92)
Low cholesterol	174	98 (0.94–1.02)	78 (0.70–0.86)	31 (0.23–0.39)	100 (0.98–1)	4.5	0.0	80 (72–86)
Elevated bilirubin	7.3	88 (0.79–0.97)	50 (0.40–0.60)	15 (0.12–0.18)	98 (0.95–0.99)	1.8	0.2	53 (45–62)
Elevated LDH	58	100 (1–1)	37 (0.28–0.46)	14 (0.12–0.16)	100 (1–1)	1.6	0.0	43 (35–51)
Fever ≥ 38.5 °C	45	92 (0.86–0.98)	79 (0.71–0.87)	30 (0.23–0.39)	99 (0.98–1)	4.4	0.1	80 (74–86)
Triad: fever, vomiting, chills	47.5	71 (0.61–0.8)	95 (0.91–0.99)	59 (0.37–0.77)	97 (0.96–0.98)	14.3	0.3	93 (88–96)
Set of 5 lab predictors	217	52 (0.37–0.66)	100 (0.96–1)	100 (1–1)	96 (0.94–0.97)	–	0.5	96 (91–98)

The positive predictive value (PPV), negative predictive value (NPV) and accuracy were calculated for a disease prevalence of 9%

*Triad of fever ≥ 38.5 °C, vomiting, and chills* The frequency of this combination of symptoms differed significantly between the two groups (*p* < 0.01). These symptoms accounted for the complaints of the majority of patients upon seeking medical care, i.e., 55/77 = 71% in the malaria group vs. 5% in the non-malaria group (*p* < 0.01). There was a positive association between the *triad of fever ≥ 38.5 °C, vomiting, and chills* and a diagnosis of malaria (OR = 47.5, *p* < 0.01) (Table 4).

### Association of laboratory parameters with malaria diagnosis

The laboratory parameters listed in Table 3 showed statistically significant differences in the mean serum concentrations between the malaria and non-malaria groups (*p* < 0.01). These parameters were then further evaluated for their association with a diagnosis of malaria. The cutoff values used in this study were chosen based on the clinical relevance, guided by the normal reference ranges used in hospital laboratories, or were estimated by calculating the likelihood ratios in the study participants.

*Thrombocytopenia* Patients with malaria had a lower mean platelet count than patients with other diagnoses (*p* < 0.01, 95% CI 97.50, 134.57). Among patients with malaria, 71% (55/77) had thrombocytopenia < 150 × 10^9^/L compared to 11% of patients with other diagnoses (*p* < 0.01). Eighty-six percent of patients with *P. vivax* infections, 52% with *P. falciparum* infections, and all patients with mixed *P. falciparum* and *P. vivax* infections developed thrombocytopenia (*p* > 0.01). There was a positive association between the presence of thrombocytopenia (<150 × 10^9^/L) and a diagnosis of malaria (OR = 20, *p* < 0.01) (Table 4). The present results showed a negative correlation between parasite density and platelet count (Table 5), whereas a positive correlation was found between the duration of the disease (before and after treatment) and the platelet count (Table 6).

**Table 5:** Platelet numbers associated with different parasite densities

Parasite index (%)	Mean platelet count
0.01–0.09	123.8
0.1–0.9	127
1–5	132
>5	92

The correlation coefficient is *r* = −0.2, *p* = 0.8

**Table 6:** Platelet numbers on consecutive days after admission

Day	Platelet mean (×10^9^/L)
Before treatment	125.4
1st day of treatment	111
2nd day	130
3rd day	143.5
4th day	245
5th day	357.5

Spearman’s test yielded a correlation coefficient of *r* = 0.943. A positive correlation was significant at the 0.01 level (*p* = 0.002)

*Hypocholesterolemia* Patients with malaria had a lower mean cholesterol level than patients without malaria (*p* < 0.01, 95% CI 1.75, 2.31). Ninety-eight percent of the malaria patients (49/50) in this study had hypocholesterolemia < 3 mmol/L vs. 22% of the patients with other diagnoses (*p* < 0.01). There was a positive association between the presence of hypocholesterolemia (<3 mmol/L) and a diagnosis of malaria (OR = 174, *p* < 0.01) (Table 4).

*Elevated LDH* Patients with malaria had a higher mean LDH concentration than patients with other diagnoses (*p* < 0.01, 95% CI −198.1, −123.1). In all malaria patients tested for LDH (50), the value was >190 mmol/L, whereas this elevation was observed in 63% of non-malaria patients (*p* < 0.01). There was a positive association between the presence of serum LDH > 190 mmol/L and a diagnosis of malaria (OR = 58, *p* < 0.01) (Table 4).

*Hyperbilirubinemia* Patients with malaria had a higher mean bilirubin level than patients with other diagnoses (*p* < 0.01, 95% CI −44.94, −24.71). Among malaria patients, serum total bilirubin was >17 μmol/L in 88% (44/50) and consisted mainly of unconjugated bilirubin, vs. 50% of patients with other diagnoses (*p* < 0.01). There was a positive association between a serum bilirubin concentration > 17 μmol/L and a diagnosis of malaria (OR = 7.3, *p* < 0.01) (Table 4).

*Low white blood cell (WBC) count* Malaria patients had a lower WBC count than patients with other diagnoses (*p* < 0.01, 95% CI 4.11, 6.39). In the former group, lymphocytes were predominant, whereas in the latter, neutrophils were predominant (*p* < 0.01). Sixty-nine percent of the malaria patients (53/77) had a WBC ≤ 8 × 10^9^/L vs. 38% of those with other diagnoses (*p* < 0.01). There was a positive association between a WBC ≤ 8 × 10^9^/L and a diagnosis of malaria (OR = 3.6, *p* < 0.01) (Table 4).

#### Set of five laboratory predictors

A set of five laboratory parameters, namely WBC < 8 × 10^9^/L, thrombocytopenia < 150 × 10^9^/L, hypocholesterolemia < 3 mmol/L, hyperbilirubinemia > 17 μmol/L, and elevated LDH > 190 U/L was assessed for its association with malaria. Fifty-two percent of malaria patients (26/50) had positive findings for all five laboratory parameters vs. 0% of patients with other diagnoses (*p* < 0.01). There was a positive association between positive findings for the full set of laboratory parameters and a diagnosis of malaria (OR = 228.5, *p* < 0.01) (Table 4).

### Diagnostic performance of clinical and laboratory parameters in malaria diagnosis

Based on the findings of the gold-standard thin and thick blood film examination as a reference test, the diagnostic accuracy of the clinical and laboratory parameters that demonstrated associations with malaria was evaluated in 77 patients with malaria and 100 nonmalaria patients for clinical parameters, and in 50 patients with malaria and 100 nonmalaria patients for laboratory parameters. Only these individuals had finished all of the clinical and laboratory tests needed for this study. Patients with symptoms suggestive of malaria were tested in a blinded manner for the reference test, and for the clinical and laboratory parameters as index tests. The clinical and laboratory parameters included in this assessment were fever ≥ 38.5 °C; the triad of fever ≥ 38.5 °C, vomiting, and chills; WBC < 8 × 10^9^/L; thrombocytopenia < 150 × 10^9^/L; hypocholesterolemia < 3 mmol/L; hyperbilirubinemia > 17 μmol/L; elevated LDH > 190 U/L; and the set of five laboratory parameters.

The assessment of diagnostic accuracy included test sensitivity, specificity, PPV, NPV, +LR, −LR, and accuracy tests. The results for the diagnostic performance of each suggested clinical and laboratory parameter, and for combinations of parameters, are shown in Table 4.

## Discussion

This study aimed to evaluate the utility of simple clinical and laboratory findings in predicting malaria infection and aiding the diagnosis of malaria in nonendemic settings. I identified a combination of clinical and laboratory markers that can reliably predict malaria infection, thus improving diagnostic accuracy and facilitating timely and appropriate treatment (Tables 2, 3, 4).

### Clinical predictors

The clinical triad of fever ≥ 38.5 °C, vomiting, and chills was strongly associated with malaria (OR = 47.5) and exhibited high accuracy (93%), specificity (95%), PPV (59%), and +LR (14.3). However, its low sensitivity (71%) limits its use as a standalone diagnostic tool as it may not always be present in all patients with malaria. Previous studies have identified additional clinical features, such as abdominal pain, myalgia, sweating, splenomegaly, and poor general health, as potential indicators of malaria. For example, Ansart et al. [18] reported correlations between malaria and abdominal pain, vomiting, and myalgia in febrile travelers returning from tropical countries. Taylor et al. [19] identified fever and splenomegaly as factors that increase the likelihood of malaria. In addition, D’Acremont et al. [3] reported that sweating, no abdominal pain, fever ≥ 38 °C, an enlarged spleen, and poor general health are associated with malaria. While these clinical findings can aid in clinical suspicion, the reliance on specific symptoms can lead to missed diagnoses in atypical presentations; hence, they should be interpreted alongside other diagnostic tests for a definitive diagnosis.

### Laboratory predictors

The following laboratory parameters showed statistically significant differences between patients with malaria and those with other diagnoses (*p* < 0.01):

Hypocholesterolemia was strongly associated with malaria in the present study (OR = 174) and demonstrated high sensitivity (98%) and negative predictive value (100%). However, its lower specificity, positive predictive value, and positive likelihood ratio led to a high rate of false positive results. A previously conducted meta-analysis [20] revealed significant reductions in total cholesterol, LDL, and HDL in malaria patients compared to controls, along with elevated triglycerides. Thirty out of 36 studies reported lower total cholesterol in malaria patients, while two studies reported elevated levels [21, 22], three found no significant differences [23–26], and one was inconclusive [26]. A more recent study in 2017 revealed hypocholesterolemia in 80% of cases, hypertriglyceridemia in 37%, and decreased HDL in 93% [27]. The diagnostic utility of hypocholesterolemia for malaria has been a subject of debate. While descriptive studies have reported high sensitivity (over 75%) for imported malaria, a case‒control study reported lower sensitivity (40%) and higher specificity (98%) [28–30]. The inconsistencies in the literature may be due to variations in study design, population characteristics, and parasite intensity. The mechanism underlying the association between hypocholesterolemia and malaria remains unclear. It has been hypothesized that the inflammation and oxidative stress associated with malaria infection may contribute to lipid metabolism disturbances.

Thrombocytopenia was strongly associated with malaria in the present study (OR = 20) and exhibited high accuracy (87%) and specificity (89%). However, its lower sensitivity limits its diagnostic utility as a standalone test. Previous studies have reported conflicting results regarding the association between thrombocytopenia and malaria [30]. Ansart et al. [18] reported an association between thrombocytopenia and malaria. Gjørup et al. [31] reported lower platelet counts in malaria patients than in nonmalaria patients. Khermach et al. [27] found thrombocytopenia in up to 90% of malaria cases. Other studies [3, 32] reported that thrombocytopenia was the factor with the highest likelihood ratio for malaria diagnosis. Badiaga et al. [29] found thrombocytopenia to be the most sensitive parameter, despite its poor specificity. The discrepancies among studies may be due to factors such as the predominant *Plasmodium* species, parasite density, response to therapy, and disease duration. In the present study, thrombocytopenia was more common in *P. vivax* infections than in *P. falciparum* infections. However, other studies have reported thrombocytopenia in both *P. falciparum* and *P. vivax* infections [33, 34]. The current study revealed a negative correlation between parasite density and platelet count (Table 5), whereas a positive correlation was observed between disease duration and platelet count (Table 6). Thrombocytopenia in malaria can be attributed to several factors, including splenic sequestration, platelet destruction, and impaired platelet production. The severity of thrombocytopenia may vary depending on the parasite species, parasite load, and host immune response.

Leukopenia was less strongly associated with malaria and exhibited limited diagnostic performance in the present study (OR = 3.6) with an accuracy of 63%. While it demonstrated low sensitivity (69%) and negative predictive value (72%), it also showed lower specificity (62%) and positive predictive value (58%). Therefore, leukopenia may be a useful tool for ruling out malaria when negative, but it is less reliable for confirming a diagnosis and should be interpreted in conjunction with other clinical and laboratory findings. Previous studies have reported conflicting findings regarding the association between leukopenia and malaria. A meta-analysis by Kotepui et al. [35] revealed that the leukocyte count was significantly lower in patients with malaria than in those without malaria and those with other febrile diseases. However, no significant difference was found between patients with malaria and healthy controls. D’Acremont et al. [3] reported that a leukocyte count ≤ 10 × 103/L was associated with parasitemia. Although Badiaga et al. [29] reported a significant association between leukopenia and malaria, they did not consider leukopenia a predictor based on their multivariate analysis. Factors such as age, *Plasmodium* species, secondary infections, and parasite load may influence leukocyte counts in malaria patients [36, 37]. The mechanisms underlying leukopenia in malaria patients are complex and not fully understood. Several factors may contribute to leukopenia, including splenic sequestration, increased cell destruction by parasites, impaired bone marrow function, and cytokine-mediated effects.

Elevated bilirubin levels showed a weaker association with malaria (OR = 7.3) and had low diagnostic accuracy (53%). However, its high sensitivity (88%) and NPV (98%) can be useful for ruling out malaria when bilirubin levels are normal or decreased. Previous studies have reported conflicting findings regarding the association between hyperbilirubinemia and malaria. Gjørup et al. [31] reported elevated bilirubin levels in malaria patients, particularly those infected with *P. falciparum*. Kociecka et al. [38] reported elevated bilirubin values in 72% of malaria patients. Taylor et al. [19] concluded that hyperbilirubinemia had an LR of 7.3 for a diagnosis of malaria. However, Badiaga et al. [29] reported that elevated bilirubin was not significantly associated with malaria. Hyperbilirubinemia in malaria patients can result from hemolysis and impaired liver function.

Elevated LDH levels were strongly associated with malaria (OR = 58) and exhibited high sensitivity (100%). However, its low specificity (37%) and accuracy(43%) limit its diagnostic utility. A negative LDH result can be helpful in ruling out malaria, but a positive result is less specific for excluding other diseases and may not definitively confirm the diagnosis. Previous studies have reported conflicting findings regarding the association between elevated LDH and malaria. Gjørup et al. [31] reported higher LDH levels in malaria patients, particularly those with *P. falciparum* infections. Khermach et al. [27] reported high LDH levels in 53% of malaria cases. However, Badiaga et al. [29] did not consider elevated LDH to be a reliable predictor of malaria. The mechanisms underlying elevated LDH in malaria are still uncertain. Elevated LDH levels in malaria patients can be attributed to increased cell turnover, tissue damage, and impaired liver function.

#### Combined predictors

A combination of five laboratory parameters (low WBC, thrombocytopenia, hypocholesterolemia, hyperbilirubinemia, and elevated LDH) was strongly associated with malaria (OR = 217) and had high diagnostic accuracy (96%) and specificity (100%). All patients with positive findings for all five laboratory predictors were in the malaria group, with no false positive results (PPV = 100%). Hence, the presence of all five predictors together strongly suggests malarial infection and reliably rules out other diseases. However, the low sensitivity (54%) of this combination limits its ability to identify all malaria cases. Almost half of the patients with malaria were negative for the set of five predictors and did not have positive findings for one or more of the five parameters, indicating that the combination may miss some true infections. This combination may be particularly useful in nonendemic settings with low prevalence rates, where a negative result can reliably rule out malaria (NPV of 96%). Interpreting these lab predictions in the context of the clinical presentations and additional clinical findings is also advised. Further studies are needed to validate and evaluate this five-predictor scheme in various target populations and in different settings.

## Limitations and future directions

While this study provides valuable insights, it is important to acknowledge its limitations. The study population was relatively small and limited to a specific geographic region. In addition, since the study is a hospital-based study with convenience sampling, it is limited by missing data in some places, particularly for laboratory parameters, which may impact the generalizability of the results. Other confounding variables, such as the study subjects’ immune status, time spent in the hospital, underlying illnesses, immunizations, personal protection, changes in climate, behavioral patterns, and policy changes, may also have affected the study’s findings. Further validation studies in diverse populations are needed to confirm these findings. In addition, the impact of different *Plasmodium* species on the diagnostic performance of these predictors should be investigated.

In conclusion, this study highlights the potential of a combined approach using clinical and laboratory predictors to improve malaria diagnosis in nonendemic settings. While no single predictor is definitive, a judicious combination can increase diagnostic accuracy and facilitate timely and appropriate treatment, taking various interfering variables and potential overlap with other underlying conditions into consideration. A comprehensive evaluation of other clinical data and other relevant factors is necessary to avoid misdiagnosis. Further research is needed to elucidate the mechanisms underlying the associations between these biomarkers and malaria and to determine their optimal use as diagnostic biomarkers. Future research should focus on validating these findings in larger, diverse populations, and exploring additional biomarkers that may further improve diagnostic accuracy.

## Conclusions

The world has made great strides toward beating malaria; however, no single tool alone is likely to end this disease. Improving diagnostic methods is a key factor in efforts to eliminate this deadly disease and ensure that it does not reappear. The diagnosis of imported malaria may constitute a challenge to healthcare providers who have less experience with the disease and its diagnostic tools. Clinical assessment can provide great diagnostic benefits, and the development of a validated simple set of diagnostic clinical features and laboratory results may provide a useful opportunity to increase our ability to predict the diagnosis.

In the present study, specific clinical and laboratory findings were shown to be significantly associated with a diagnosis of malaria, and can thus together be considered potentially useful as a helpful clinical tool. These predictors were fever ≥ 38.5 °C, vomiting, and chills, together with leukopenia < 8 × 10^9^/L, thrombocytopenia < 150 × 10^9^/L, hypocholesterolemia < 3 mmol/L, hyperbilirubinemia > 17 μmol/L, and elevated LDH levels > 190 U/L.

A simple approach based on the set of clinical and laboratory parameters proposed here could alert clinicians to the possibility of malaria and help practitioners in nonendemic areas avoid overlooking malaria as the likely diagnosis. The judicious application of such clinical and laboratory predictors, while considering potential overlap with other underlying conditions, may improve case detection and enhance the diagnosis of imported malaria cases in nonendemic settings. For febrile patients who have recently returned from a malaria-endemic area but who have a negative thin and thick blood smear, it may be helpful to consider the clinical and laboratory predictors analyzed here as suggestive of malarial infection. In patients with positive findings for these predictors, careful reevaluation or repetition of blood smears may be advisable. However, rigorous validation of this set of predictors in diverse populations is crucial before its widespread clinical application. Further validation studies are now needed to evaluate this diagnostic method in different settings.

## Data Availability

No datasets were generated or analyzed during the current study.
